# A Shifting Trend Towards Endovascular Intervention in the Treatment of Acute Mesenteric Ischemia

**DOI:** 10.7759/cureus.18544

**Published:** 2021-10-06

**Authors:** Ali A Naazar, Ahmad Omair, Samuel H Chu, Kevin G Keane, Dieter G Weber

**Affiliations:** 1 General Surgery, Royal Perth Hospital, Perth, AUS; 2 Pathology, College of Science & Health Professions, King Saud bin Abdulaziz University for Health Sciences & King Abdullah International Medical Research Center, Riyadh, SAU

**Keywords:** mortality, treatment outcome, surgery, endovascular intervention, acute mesenteric ischemia

## Abstract

Background

Acute mesenteric ischemia (AMI) is a vascular emergency with a quite low incidence, but it is associated with disproportionately more severe morbidity and mortality. The aim of this study was to assess the current trend in the treatment of AMI and to see if endovascular intervention is an effective treatment modality in the selected group of patients.

Methods

A retrospective review of patients admitted with AMI between 2007 and 2018 was performed. Outcome measures were length of stay (LOS) at hospital and intensive care unit (ICU), and post-treatment mortality.

Results

A total of 98 patients with AMI were admitted during the study period. Patients undergoing endovascular treatment compared with surgery were younger (62.9 ± 13.7 years vs*.* 69.5 ± 12.8 years; p = 0.01). Shorter LOS in hospital and ICU was observed for those treated with endovascular approach (6.8 ± 3.4 and 3.25 ± 0.5 days) compared to the surgical group (25 ± 8.6 and 12.8 ± 26.8 days; p < 0.001). Out of 39 patients requiring ICU admission, 48.7% were surgically treated and 10.2% underwent endovascular intervention (p < 0.001). Mortality associated with surgery was 30.6% compared to only 6.6% with endovascular intervention (p < 0.001). Between 2007 and 2012, only one patient underwent endovascular intervention and 20 underwent surgery compared to 14 patients treated with endovascular approach and 16 with surgery between 2013 and 2018.

Conclusion

In this non-randomized, retrospective case series, patients with endovascular treatment fared clinically better and the intervention was found to be safe and feasible in the selected group of patients. We suggest a preference for this modality where possible. At our hospital, a trend favoring this approach is apparent during the last six years.

## Introduction

Acute mesenteric ischemia (AMI) is a vascular emergency involving ischemia of the small bowel, with associated inflammation and potentially infarction and necrosis [1]. AMI is an uncommon entity with a quite low incidence reported to be 0.09% to 0.2% of all acute surgical conditions presented in the emergency [1-3]. However, it is associated with disproportionately more severe morbidity and mortality. Other studies report mortality and morbidity between 16% and 80% [4-6] and 25% and 100% [7-10]. Bowel ischemia could be a result of arterial thrombosis (25%), venous thrombosis (15%), and arterial embolism (50%) [11].

Despite advancements in medical science, the diagnosis of AMI remains a challenge owing to it being an uncommon cause for abdominal pain and a low incidence rate [2,12]. The presentation is heterogeneous, mainly presenting with abdominal pain that could be associated with nausea, vomiting, diarrhea, and hematochezia [13]. A high degree of clinical suspicion is required to make an early diagnosis [14]. Due to nonspecific symptoms and unavailability of any reliable diagnostic test outside imaging, the diagnosis is often delayed and could even lead to the death of the patient. Computed tomography angiography (CTA) has been recommended even in patients in whom nephrotoxicity concerns from iodinated contrast media exist, owing to high specificity and sensitivity of CTA in predicting AMI [15]. Unfortunately, delays in diagnosis can compound the clinical situation to a point beyond the availability of meaningful endovascular interventions without the addition of open surgery for necrotic consequences in the bowel.

Based on the cause of occlusion, surgical revascularization may involve embolectomies, angioplasty, and/or bypass procedures. On the other hand, endovascular techniques have also been becoming popular in revascularization of the vessel. So far, no randomized control trial has been performed to assess and compare open surgery and endovascular approach, as to which of the two treatment modalities should be the primary management strategy [16]. A systematic review revealed no significant difference in treatment outcome by surgery or vascular bypass [17]. Much controversy surrounds the use of endovascular techniques as primary management of AMI [18]. On one hand, studies have reported a lesser need for laparotomy, a shorter length of bowel resection, and a significantly lower mortality rate with endovascular techniques compared to surgery [19]. On the other hand, endovascular techniques could only be used before the ischemia leading to infarction, could involve many contraindications, and are often followed by laparotomy. Contrary to this, open surgery is effective in assessing the viability of the bowel and hence preventing delay in revascularization [1].

This study aimed to assess the current practice in the management of AMI in an Australian tertiary hospital. We further aimed to assess the trends in the management of AMI between 2007 and 2018 at our hospital and to assess the outcome between patients treated with different treatment modalities. We hypothesized that there is a definite change in practice regarding the increased use of the endovascular first approach in the management of AMI and that this is associated with better patient outcomes in the selected group of patients.

This is an original research study and was previously presented as an abstract at the 2021 RACS 89th Annual Scientific Congress, Melbourne, Australia, on May 14, 2021.

## Materials and methods

Study sample and design

A retrospective query of Royal Perth Hospital’s coding database was performed to identify patients admitted with acute bowel ischemia between 2007 and 2018. Subsequent data collection was conducted by review of the medical record. Demographics and outcomes were abstracted from the medical record.

Exclusion criteria

Patients with large bowel ischemia, venous thrombosis, and bowel ischemia due to incarcerated hernia were excluded from this study. Only patients with small bowel ischemia due to arterial thrombosis or embolism were included.

Diagnosis and treatment modalities

Diagnosis of AMI was pragmatic, as recorded and confirmed clinically at the time of care, based on clinical history, examination, CTA, and laparotomy findings. Endovascular treatment involved balloon dilatation and stent implantation. Patients who were hemodynamically stable and without peritonitis on clinical examination and for whom CT scan revealed bowel ischemia without infarction and/or perforation were chosen for endovascular treatment. In some cases, vascular surgery was accompanied by open surgical treatment. Open surgery involved arterial embolectomy, laparotomy, excision of the necrotic bowel, and delayed abdominal closure. Some cases were treated only by non-operative management, and some had palliative treatment where other therapies were contraindicated.

Data analysis

Data are presented as counts and percentages for categorical variables and as mean ± standard deviation (SD) for the continuous variables. The demographic characteristics abstracted included age, gender, and ethnicity. Data regarding any comorbidity, use of anticoagulants, and use of CTA for diagnosis were also recorded. The outcome was measured by the total length of stay (LOS) at the hospital, length of intensive care unit (ICU) stay, and whether the patient was alive or dead post-treatment. Stratification for the purpose of the descriptive analysis was performed using Microsoft Excel. OpenEpi was used to calculate p-values for difference among groups using the chi-square test for variables given in percentages and ANOVA (analysis of variance) for variables given as mean. Fisher’s exact test was performed for comparison of dead and alive patients among five treatment groups. A test with a p-value of <0.05 was considered statistically significant.

Ethics

The study was approved by the Research Ethics Committee at the Royal Perth Hospital, Perth, Australia (Ref: CSQU236).

## Results

A total of 98 patients were identified for the study. Mean age of our study sample was 70.5 years (SD = 14.7) and ranged between 28 to 94 years. Out of the 98 patients, 57 (58.2%) were males and 41(41.8%) were females. Most (56.1%) were nonindigenous, 41% whose ethnicity was not known, and 2.1% were indigenous (Table 1).

**Table 1:** Demographic characteristics of 98 AMI patients stratified according to different treatment therapies AMI, acute mesenteric ischemia

Patient characteristics	Total, N (%)	Treatment type					p-Value
		Endovascular, N (%)	Surgical, N (%)	Vascular + surgical, N (%)	Non-operative, N (%)	Palliative, N (%)	
Total number of cases in the cohort	98 (100)	15 (15.3)	36 (36.7)	11 (11.2)	20 (20.5)	16 (16.3)	-
Age, years (mean ± SD)	70.5 ± 14.7	62.9 ± 13.7	69.5 ± 12.8	64.6 ± 12.3	77.6 ± 13.6	75.1 ± 18.2	0.01
Age, years (range)	28-94	44-83	39-86	48-83	46-91	28-94	-
Gender							
Males, % of total (58.2)	57 (100)	12 (21.1)	21 (36.8)	5 (8.8)	10 (17.5)	9 (15.8)	0.37
Females, % of total (41.8)	41 (100)	3 (7.3)	15 (36.6)	6 (14.6)	10 (24.4)	7 (17.1)	
Ethnicity							
Nonindigenous, % of total (56.1)	55 (100)	11 (20)	20 (36.3)	6 (10.9)	9 (16.4)	9 (16.4)	0.59
Indigenous, % of total (2.1)	2 (100)	0 (0)	1 (50)	1 (50)	0 (0)	0 (0)	
Unknown, % of total (41.8)	41 (100)	4 (9.8)	15 (36.5)	4 (9.8)	11 (26.8)	7 (17.1)	

Among the 98 patients, 15 (15.3%) underwent endovascular intervention, another 11 (11.2%) were intervened with the vascular surgical approach followed by open general surgery, and 36 (36.7%) were only surgically treated; 20 (20.5%) patients underwent non-operative management and 16 (16.3%) were palliated. Twice as many patients were surgically treated compared to endovascular approach.

Relatively younger patients underwent either endovascular treatment or vascular surgery followed by surgical treatment (62.9 ± 13.7 and 64.6 ± 12.3, respectively) compared to the surgical patients whose mean age was 69.5 ± 12.8 years. Patients treated non-operatively and those who received palliative treatment were the oldest in our cohort (77.6 ± 13.6 and 75.1 ± 18.2, respectively). There was a statistically significant difference in the age of patients among different treatment groups (p = 0.01).

Four times as many males underwent endovascular treatment compared to females, whereas the tendency for surgical treatment was only 1.5 times more in males. No statistically significant difference was observed among males and females among the treatment groups (p = 0.37).

Only two patients were indigenous, out of whom none underwent endovascular intervention. Approximately three times more non-indigenous patients (N = 11) had endovascular intervention compared to those with unknown ethnicity (N = 4). But this difference observed was not found to be statistically significant (p = 0.59).

Table 2 presents the clinical characteristics of the 98 patients stratified according to treatment type. The average LOS in the hospital for our cohort was 16.7 ± 22.5 days. A statistically significant difference (p < 0.001) was observed in the LOS among the different treatment groups, with the shortest stay observed for those treated with endovascular approach (6.8 ± 3.4 days) and the longest stay for the surgical group and patients treated with vascular surgical intervention followed by open surgery (25 ± 8.6 and 27 ± 20 days, respectively). The LOS for patients managed non-operatively was 10.9 days and for those palliated it was 12.8 days.

**Table 2:** Diagnosis and outcome of 98 AMI patients stratified according to different treatment therapies ICU, intensive care unit; CTA, computed tomography angiogram *40 patients did not need ICU admission, and for 19 patients, data were not available.

Outcome	Total N (%)	Treatment type					p-Value
		Endovascular, N (%)	Surgical, N (%)	Vascular + surgical, N (%)	Non-operative, N (%)	Palliative, N (%)	
Number of cases	98 (100)	15 (15.3)	36 (36.7)	11 (11.2)	20 (20.5)	16 (16.3)	-
Length of stay days, mean ± SD	16.7 ± 22.5	6.8 ± 3.4	25 ± 8.6	27 ± 20	10.9 ± 16.6	7.7 ± 16.8	<0.001
Number of cases admitted to ICU, N (%)*	39 (100)	4 (10.2)	19 (48.7)	9 (23.1)	1 (2.6)	6 (15.4)	<0.001
ICU stay in days, mean ± SD	10.4 ± 20.8	3.25 ± 0.5	12.8 ± 26.8	7.7 ± 5.6	1	12.8 ± 23.4	0.15
Alive/dead							
N	69/29	14/1	25/11	8/3	20/0	2/14	<0.001
%	70.4/29.6	93.4/6.6	69.4/30.6	72.7/27.3	100/0	12.5/87.5	
Comorbidity (N)							
Yes	85	12	31	9	18	15	0.79
No	9	2	4	2	0	1	
Unknown	4	1	1	0	2	0	
Anticoagulants (N)							
Yes	59	12	18	8	14	7	0.12
No	33	3	15	3	5	7	
Unknown	6	0	3	0	1	2	
CTA, N (%)							
Yes	67 (68.4)	11 (73.3)	22 (61.1)	9 (81.8)	15 (75)	10 (62.5)	0.62
No	31 (31.6)	4 (26.7)	14 (38.9)	2 (18.2)	5 (25)	6 (37.5)	

A total of 39 patients required ICU admission, with an average LOS in ICU of 10.4 ± 20.8 days. ICU admission was required for 10.2% of patients treated by endovascular intervention and 9% who were treated both by vascular and open surgery. However, among the patients requiring ICU admission, almost half of them (48.7%) were treated surgically. Only one patient treated non-operatively and six among the palliative group required ICU admission. There was a statistically significant difference among patients requiring ICU admission from different treatment groups (p < 0.001). The LOS in ICU was again shorter among the endovascular group (3.25 ± 0.5 days), followed by those who were treated both by vascular and open surgical approach (7.7 ± 5.6 days). Compared to these, the surgically treated patients required almost four times longer (12.8 ± 26.8 days) stay in ICU (Table 2). But this difference was not found to be statistically significant (p = 0.15).

Among our cohort of 98 patients, 69(70.4%) were alive and discharged, whereas 29 (29.6%) died. None among non-operatively managed patients and 87.5% of palliated patients died. On the other hand, 30.6% of surgically treated patients, 6.6% of those who underwent endovascular intervention, and 27.3% of those who received both vascular and open surgery died. The mortality rate in surgical patients was almost five times higher than the endovascular group and was found to be statistically significant (p < 0.001).

Among our cohort, 85 patients had comorbidity and only nine had no comorbidity, whereas data regarding comorbidity were not available for four patients. The prevalence of comorbidity was not substantially different among the endovascular group, surgical group, and those treated with vascular surgery followed by open surgery (80%, 86%, and 82%, respectively). Contrary to these, 90% of those treated non-operatively and 94% of those palliated had comorbidities (Table 2). The difference among groups was not statistically significant (p = 0.79).

CTA was used for the diagnosis of AMI in 67 (68.4%) patients in our cohort. The lowest percentage of patients who underwent CTA were among the surgical group (61.1%) followed by the palliative group (62.5%); 73.3% of patients treated with endovascular intervention and 75 % treated non-operatively underwent CTA. The highest percentage of patients who underwent CTA were those treated with both vascular and open surgical approach (81.8%). No statistically significant difference was found in the use of CTA among different treatment groups (p = 0.62).

The yearly trend of different treatment types for 98 AMI patients from 2007 to 2018 is given in Table 3.

**Table 3:** Year-wise trend of different treatment modalities among 98 patients diagnosed with AMI from 2007 to 2018 AMI, acute mesenteric ischemia

	Treatment type					
Year	Endovascular, N (%)	Surgical, N (%)	Vascular + surgical, N (%)	Non-operative, N (%)	Palliative, N (%)	Total, N (%)
2007	1 (14.3)	5 (71.4)	0 (0)	0 (0)	1 (14.3)	7 (100)
2008	0 (0)	6 (75)	0 (0)	1 (12.5)	1 (12.5)	8 (100)
2009	0 (0)	3 (50)	0 (0)	3 (50)	0 (0)	6 (100)
2010	0 (0)	2 (28.6)	1 (14.3)	1 (14.3)	3 (42.8)	7 (100)
2011	0 (0)	1 (20)	1 (20)	3 (60)	0 (0)	5 (100)
2012	0 (0)	3 (50)	0 (0)	1 (16.7)	2 (33.3)	6 (100)
2013	3 (25)	4 (33.3)	2 (16.7)	2 (16.7)	1 (8.3)	12 (100)
2014	2 (11.8)	3 (17.7)	4 (23.5)	5 (29.4)	3 (17.6)	17 (100)
2015	2 (40)	1 (20)	1 (20)	0 (0)	1 (20)	5 (100)
2016	2 (28.6)	3 (42.8)	2 (28.6)	0 (0)	0 (0)	7 (100)
2017	3 (33.3)	2 (22.2)	0 (0)	1 (11.2)	3 (33.3)	9 (100)
2018	2 (22.2)	3 (33.3)	0 (0)	3 (33.3)	1 (11.2)	9 (100)
Total N (%)	15 (15.3)	36 (36.7)	11 (11.2)	20 (20.5)	16 (16.3)	98 (100)

**Figure 1:**
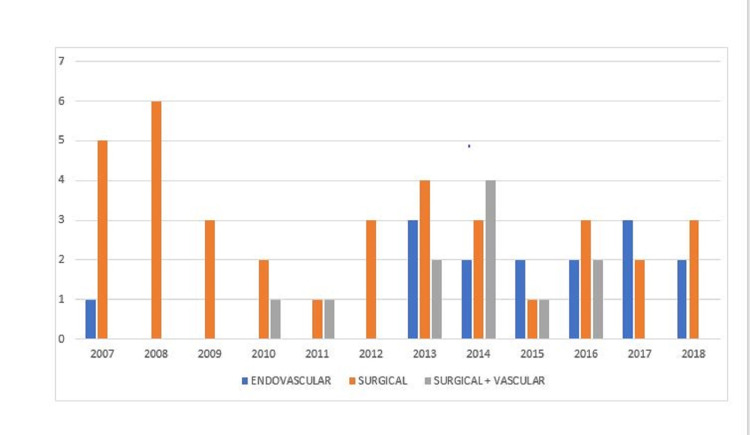
A bar diagram showing trends of endovascular, surgical, and vascular plus surgical intervention between 2007 and 2018

We observed that patient load increased at our center as out of 98 patients treated over a period of 12 years, 59 were treated in the last six years (2013 to 2018) compared to 39 patients in the first six years (2007 to 2012). Only one patient out of these 39 patients underwent endovascular intervention in the first six years compared to 14 patients in the last six years. Similarly, only two patients were treated by vascular surgery followed by open surgery in the first six years compared to nine in the last six years. The number of surgically treated patients on the other hand decreased in the last six years (N = 16) compared to the first six years (N = 20). In the year 2015, there were more patients treated with endovascular treatment (40%) compared to surgery (20%) and other groups as well. A similar trend was observed in 2017, when 33.3% of patients underwent endovascular intervention compared to surgery (22.2%) (Figure 1).

## Discussion

AMI continues to present in a heterogenous fashion and remains associated with significant morbidity and mortality. During this 12-year period of this review, the endovascular intervention appears to have become more common when compared with open surgical options at our center. We hypothesize that this change may reflect the more favorable procedural risk profile of such an approach, as well as improvements in diagnosis and imaging accuracy, though the current study was not designed to address these clinical decisions.

In a recent experience elsewhere, Beaulieu et al. reported higher use of endovascular intervention (24% of their patients) in a large case series of 679 patients [4]. Interestingly, these authors also reported a higher percentage (20.5%) of patients who were managed non-operatively. We wonder if this different course may be explained by different clinical or geographic systems and factors, which facilitate earlier diagnoses, thereby facilitating endovascular options and avoiding the need for surgical resections of the intestine. The percentage of the surgically treated patients remains high because a large number of AMI patients continue to present as a surgical emergency with frank peritonitis and/or bowel ischemia excluding a pure endovascular approach [20]. Although endovascular treatments appear to have become more popular, comparison between open surgery and endovascular approach has not been assessed in a randomized controlled fashion [16].

Mortality rates from AMI in excess of 50% remain common in the literature [5,21]. While our experience is somewhat more favorable (29.5%), more information regarding the aggressiveness of treatment strategies, and palliative decisions among the various published experiences, is required to meaningfully compare this statistic between centers. In our experience, the mortality in AMI is mainly attributed to multi-system organ failure related to bowel necrosis and associated sepsis, classically associated with late diagnoses [1], rather than definable postoperative complications or pathological issues with the underlying vascular disease.

Assuming the need for surgery represents a surrogate marker for advanced pathology, and not unsurprisingly Beaulieu et al. report lower mortality in their endovascular patients compared with their surgical cohort (25% vs. 40%) [4]. Similarly, Schermerhorn et al. reported a mortality rate of 16% in patients treated with the endovascular intervention compared to 28% among surgically treated [6]. However, this observation could also be explained by increased peri-procedural complications associated with open surgery. Further analyses of the cause of the excess mortality associated with open surgery would assist this discussion and remain beyond the scope of the current series.

CTA remains the key tool in early and accurate diagnosis of AMI, with reported high specificity and sensitivity (97.9% and 93%, respectively) [15]. In our experience, we see a relation between the use of CTA, the use of endovascular intervention, and lower mortality. Most of the patients treated with open surgery presented as a vascular emergency and underwent laparotomy for better visualization of the bowel and its resection. Therefore, CTA was least used in this group.

The average LOS for patients with open surgery had been found to be longer than that in the endovascular cohort - observed both in this series and by authors elsewhere. Compared with Beaulieu et al. (12.9 days for the endovascular group vs. 17.1 days for the surgical group) [4], our results are more distinct, perhaps by an effect of patients with advanced pathologies; at the time of the study, the hospital served as the dominant referral center for the rural population of Western Australia, translating to more pronounced delays in these patients’ diagnoses. Unfortunately, insufficient data regarding the location of the initial diagnosis and prehospital retrieval times were available to make a meaningful analysis.

The current study presents a significant long-term retrospective analysis of Australian patients in a geographically isolated state. However, the study is limited by the relative infrequency of the clinical event and the resultant small sample size. Another significant limitation of this study is the selection bias as relatively young and less sick patients, without bowel necrosis, were selected for the endovascular approach, which could have resulted in relatively better outcomes. The study is further limited by the inherent difficulties in the retrospective application of various clinical definitions and the accuracy of the medical record.

## Conclusions

We conclude that endovascular treatment is more attractive among selected AMI patients. However, whether the improved benefits associated with this approach stem from more favorable underlying pathology, from the approach itself, from a combination of these, or from factors not yet considered requires further investigation. We suggest that some of the benefits may relate to earlier diagnosis and that this remains central in the good management of AMI. The utility of CTA as a key tool in the diagnosis of AMI is reinforced in this series. We suggest that a multidisciplinary team approach involving better coordination among general/vascular surgeons, radiologists, intensivists, and anesthetists can lead to a better selection of patients who can be treated with endovascular first approach to achieve maximum benefit.
